# Supplementary material for: The Systems Biology Research Tool: evolvable open-source software
Source: BMC Syst Biol. 2008 Jun 29;2:55. doi: 10.1186/1752-0509-2-55 (PMC2446383; doi:10.1186/1752-0509-2-55)
Supplement: Additional file 1 — SBRT Archive. An archive of the current version of the Systems Biology Research Tool. [file 1752-0509-2-55-S1.zip › sbrt-1.4.0/doc/developers_guide/api/sbrt/kernel/io/package-frame.html]

sbrt.kernel.io


sbrt.kernel.io

Interfaces 
  
*ResultsManager*
  
*WarningHandler*

Classes 
  
AbstractResultsManager
  
ListResultsManager
  
SingleRm
  
WarningHandlerFactory

Exceptions 
  
ConflictingInputException
